# Assessment of Knowledge and Attitude among Pharmacists toward Pharmaceutical Care in Eastern Ethiopia

**DOI:** 10.1155/2020/7657625

**Published:** 2020-02-17

**Authors:** Kirubel Minsamo Mishore, Abraham Nigussie Mekuria, Assefa Tola, Yohanes Ayele

**Affiliations:** ^1^Department of Clinical Pharmacy, School of Pharmacy, College of Health and Medical Sciences, Haramaya University, Harar, Ethiopia; ^2^Department of Pharmacology, School of Pharmacy, College of Health and Medical Sciences, Haramaya University, Harar, Ethiopia; ^3^Department of Epidemiology and Biostatistics, School of Public Health, College of Health and Medical Sciences, Haramaya University, Harar, Ethiopia

## Abstract

**Objective:**

To assess knowledge and attitudes toward pharmaceutical care service among hospital and community pharmacists working in Harar and Dire Dawa town, Eastern Ethiopia.

**Method:**

A descriptive cross-sectional study was conducted among pharmacists working in hospital and community pharmacies, 2018. A total of 43 health settings (6 hospital and 37 community pharmacies) were involved in this study. All pharmacists who met the inclusion criteria were selected using a purposive sampling technique to take part in the study. The pretested structured self-administered questionnaires were used to collect data. The collected data was coded, entered, and analyzed using Statistical Package for Social Sciences (SPSS) version 21.0. The findings were presented by frequencies and percentages, and summary measures were displayed using tables. Chi-Square test and Fisher's exact test were performed to determine the association between sociodemographic characteristics and the level of knowledge and attitude about pharmaceutical care. The study protocol was approved by the Harar Health Sciences College Research Ethics Review Committee.

**Results:**

A total of seventy-eight pharmacists were included in the study with a response rate of 97.5%. The mean age (±Standard Deviation (SD)) of the study participants was 32.47 ± 7.42 years, and the majority (88.3%) of the respondents were males. 56.4% of the respondents were working in the hospitals while 43.6% were working in community pharmacy. Overall, 85.9% of the respondents had good knowledge of pharmaceutical care. The types of training curriculum of the participants showed an association with the attitude of pharmacists (*P* value = 0.022). Similarly, pharmacists' knowledge was associated with their practice setting (*P* value = 0.022). Similarly, pharmacists' knowledge was associated with their practice setting (

**Conclusion:**

The majority of pharmacists are knowledgeable about PC. However, nearly half of the pharmacists had an unfavorable attitude toward pharmaceutical care. Harari Regional and Dire Dawa City Health Bureaus should organize and provide in-service training on pharmaceutical care to pharmacists working in community and hospital pharmacies. Furthermore, the bureaus should advocate pharmaceutical care as one area in a continuous professional development program.

## 1. Introduction

Over the past four decades, there has been a trend for pharmacy practice to move away from its original focus on medicine supply to a more inclusive focus on patient care. The role of the pharmacist has evolved from that of a compounder and supplier of pharmaceutical products to that of a provider of services and information and, ultimately, that of a provider of patient care. This new approach has been known as pharmaceutical care (PC) [1].

PC is a multifactorial and structured process that is defined per International Pharmaceutical Federation (FIP) as “the responsible provision of pharmacotherapy to achieve definite outcomes that improve or maintain a patient's quality of life” [2]. By taking direct responsibility for individual patient's medicine-related needs, pharmacists can make a unique contribution to the outcome of medical therapy and their patients' quality of life [1]. In this regard, the philosophy of PC focuses on the responsibility of the pharmacist to meet all of the patient's drug-related needs and assist the patients in achieving their goal through collaboration with other health professionals [3]. An adequate pharmaceutical service provided by the pharmacist is a vital component of the health care delivery system. Moreover, studies have reported that the implementation of PC in hospital and community settings improves patients' health outcomes, reduces hospital stay, improves medication adherence, and reduces health care costs [4–9].

In response, several professional pharmacy organizations have adopted the philosophy of PC and it was initiated in various countries to shift the demands of the pharmacy profession toward patient care [10].

In this line in Ethiopia, various efforts have been made to introduce PC in the health care system, including development and implementation of a 5-year patient-oriented Bachelor of Pharmacy (B.Pharm) curriculum in public universities since 2008 and initiating the clinical pharmacy and pharmacy practice programs at postgraduate levels [11, 12]. In addition to this, the Federal Ministry of Health (FMOH) included clinical pharmacy services in the pharmacy section of the Ethiopian Hospital Reform Implementation Guidelines (EHRIG) in 2010 [13]. Furthermore, clinical pharmacy service has been incorporated in the health facilities minimum regulatory standards by Ethiopian Standards Authority (ESA)/Ethiopian Food, Medicines and Health Care Administration and Control Authority (FMHACA) in 2012 [14].

It has been known that pharmacists' knowledge, attitudes, skill, commitments, and ethics are the foundations to provide PC to patients [3, 15]. However, several barriers have hampered the implementation of PC practice universally, including the insufficient time to provide PC, lack of pharmacists' self-confidence, inadequate clinical knowledge, and communication skills of pharmacists. Moreover, the unfavorable attitudes of pharmacists themselves toward performing PC have served as barriers to providing PC [16–18].

In this regard, because the Ethiopian pharmacy sector is experiencing PC as a new initiative, it is imperative to assess factors influencing the implementation of PC. However, as far as the knowledge of authors concerned, there is no published study about PC in the study area. Thus, this study aimed at assessing knowledge and attitudes toward PC service among hospital and community pharmacists working in Harar and Dire Dawa town, Eastern Ethiopia.

## 2. Material and Methods

### 2.1. Study Area and Period

This study was conducted among pharmacists working in community and hospital pharmacies in Harar and Dire Dawa towns, from May to June 2018. Harar and Dire Dawa towns are located in the Eastern part of Ethiopia, at a distance of 525 km and 515 km from Addis Ababa, the capital city of Ethiopia, respectively. During the study period, there were 6 hospital pharmacies (2 were private and 4 were governmental) and 16 community pharmacies in the Harar town. In these pharmacies, a total of 34 (13 community and 21 hospital) pharmacists were working and registered by the Harari regional health bureau. Similarly, there were 7 hospital pharmacies (5 were private and 2 were governmental) and 21 community pharmacies in Dire Dawa town. A total of 46 (21 community and 25 hospital) pharmacists were working in these pharmacies and registered by the Dire Dawa counsel health bureau.

### 2.2. Study Design and Study Population

A descriptive cross-sectional study was conducted among the community and hospital pharmacists working in Harar and Dire Dawa towns. All pharmacists of Harar and Dire Dawa towns were the source populations. All pharmacists working in community and hospital pharmacies of Harar and Dire Dawa towns during the study period were the study populations. Pharmacists working in a public hospital or community pharmacy and who were willing to give their informed consent were included in the study. Pharmacists who were in annual leave and sick leave were excluded from the study.

### 2.3. Sample Size and Sampling Technique

The sample for this study was all pharmacists working in community and hospital-based pharmacies of Harar and Dire Dawa towns during the study period. The list of all pharmacists was obtained from Harari Regional and Dire Dawa City Health Bureaus. Then, the list was checked to evaluate the fulfillment of the inclusion criteria. Finally, all pharmacists who met the inclusion criteria were selected using a purposive sampling technique to take part in the study.

### 2.4. Data Collection Instrument and Technique

Data was collected using a structured self-administered questionnaire which is developed after reviewing related studies from the literature [19–22]. A self-administered questionnaire was used to obtain information on sociodemographic variables, knowledge, and attitude of study participants toward PC. The questionnaire was structured into three sections: the first section was designed to collect demographic information such as the age of respondents, gender, qualification, and years of experience. The second section consisted of 10 questions to ascertain the knowledge of pharmacists about pharmaceutical care. These questions were designed using a 3-point response format consisting of “Yes,” “No,” and “I do not know.”

The third section encompassed 10 statements to assess the attitude of pharmacists toward pharmaceutical care. It is a 5-Likert-type scale (agree strongly “5”, agree slightly “4”, neutral “3”, disagree lightly “2”, and disagree strongly “1” with a total score range from 10 to 50). These statements measure three constructs: professional requirement (statements 2, 3, and 8), professional duty (statements 1, 6, 7, and 9), and professional effect (statements 4, 5, and 10). An attempt was made to balance negatively and positively worded questions to minimize mechanical responses. Therefore, five of the ten items were negatively worded [4, 6, 7, 9, 10] and the remaining five were positively worded. The five negatively worded statements were reverse-scored during the analysis so that the more positive attitudes toward PC would be reflected by higher scores.

The self-administered questionnaire was distributed by four trained and experienced pharmacies. A standardized working process was carried out by these pharmacies who distributed the questionnaire: (1) a brief self-introduction, (2) systemic explanation of the overall nature and processes of the study as indicated on the cover page of the questionnaire as participant information sheet, (3) declaration of anonymous nature of the study, and (4) confirmation of respondents' participation and taking their voluntary consent. Whenever respondents made queries about the study, the data collectors would provide more specific information. All of the questionnaires were distributed directly to the respondents at their working place and collected during the next day's visit.

### 2.5. Data Quality Control

The quality of data was ensured using various strategies like pretesting the questionnaire, training of the data collectors and supervisors, supervision, and checking the questionnaire. The data collectors and supervisors were trained on the data collection technique for two days prior to data collection processes.

After the questionnaire was developed, the content, clarity, validity, reliability, and format were evaluated using a pretest involving a convenience sample of 10 pharmacists working in private hospitals of Harar (Yimag and General Hospitals) and Dire Dawa town (Delt, Art, Bilal, and Yemaryam Work hospitals). In the pretest, pharmacists were asked to provide feedback on the design of the questionnaire, its relevance, and the flow of individual questions between sections. Comments were also obtained from three senior academic pharmacists from Haramaya University and Harar Health Science College. The pretest was also served as a practice session for the data collectors to be well acquainted with the data collection instrument.

Then, the internal consistency (reliability) of the items was tested by determining Cronbach's alpha Coefficient (minimum 0.5, ideally between 0.7 and 0.8), inspecting partial alphas of each item (>0.3), and determining the item to total correlation. After this checking, the finalized questionnaire was employed, in order to collect data from the major sample. All the collected data were checked for completeness, accuracy, and consistency by the principal investigators and the supervisors on a daily base.

### 2.6. Data Processing and Analysis

The collected data were coded, entered, and analyzed using Statistical Package for Social Sciences (SPSS) version 21.0. Descriptive statistics was used to summarize the data and organize them into sociodemographics characteristics, knowledge, and attitudes of the participants according to the sections of the questionnaires. Then, the findings were presented by frequencies and percentages, and summary measures were displayed using tables.

For knowledge questions, score 1 was assigned to the correct answers and zero was assigned to wrong answers. For attitude questions, a Likert-type summation of scores was employed. First, negatively worded questions were reversed so as to align all the scores in one direction. For the knowledge and attitude items, means and median were used to determine the overall response of the respondents, respectively. Moreover, the Chi-Square test and Fisher's exact test were performed to determine the association between sociodemographic characteristics and the level of knowledge and attitude about PC. The five factors included were gender, age, years of experience, practice setting, and training curriculum. The factors indicated statistically significant differences at the *P* < 0.05 level.

### 2.7. Ethical Consideration

The study protocol was approved by the Harar Health Sciences College Research Ethics Review Committee. A formal permission letter was obtained from Harar Health Sciences College and submitted to community and hospital pharmacy managers, and the purpose of the research and how its premise is selected were also communicated. Before the data collection, written consent was obtained from each participant by informing them of the purpose of the study.

### 2.8. Operational Definitions

#### 2.8.1. Knowledge

We used a ten-item composite score of the knowledge to measure the knowledge level of respondents regarding definition, philosophy of practice, practitioners' responsibility, goals and objectives, and roles and activities of practitioners of PC. The cumulative mean score of knowledge of participants about PC was estimated using the mean score. Based on this, those who had scored less than the mean were considered to have “poor knowledge” and those who had scored greater than or equal to the mean value were considered as having “good knowledge.”

#### 2.8.2. Attitude

To assess the respondents' attitudes toward PC, a five-point Likert scale (rating from 1 = strongly disagree to 5 = strongly agree) was utilized to measure the extent to which the respondents agreed with 10 statements related to PC. The total of each respondent score was made to range between 10 and 50. A score of median value and above was considered as a “favorable attitude” whereas those scores below median value were thought of as having an “unfavorable attitude.”

## 3. Results

### 3.1. Sociodemographic Characteristics of Study Participants

In the present study, a total of 80 questionnaires were distributed and 78 were found complete with a response rate of 97.5%. The mean age (±Standard Deviation (SD)) of the study participants was 32.47 ± 7.42 years and the majority (88.3%) of the respondents were males. Regarding training, less than half (44.9%) were trained with a clinically oriented curriculum or have got in-service training in clinical pharmacy. The respondents had an average (±SD) of 5.79 ± 5.34 years of experience. Concerning practice setting, 56.4% of the respondents were working in the hospitals while 43.6% were working in community pharmacies (Table 1).

### 3.2. Pharmacists' Knowledge of Pharmaceutical Care

As shown in Table 2, the majority of participants (94.9%) were aware of pharmaceutical care definition and purpose. A considerable proportion, 88.5% and 97.4%, of the respondents were well informed about the pharmacists' roles in pharmaceutical care delivery and the purpose of pharmaceutical care, respectively. Large parts of respondents were also informed about the primary focus of pharmaceutical care practitioners and their responsibilities such as patient assessment, planning, intervention, follow-up, and documentation during the process of service delivery. The mean score of knowledge of participants about PC was 9.0 ± 1.23. Overall, 85.9% of the respondents had good knowledge of pharmaceutical care.

### 3.3. Pharmacists' Attitude toward Pharmaceutical Care

In this assessment, over half (59%) of the respondents strongly agreed that all pharmacists should provide pharmaceutical service (Table 3). Likewise, more than half of the participants strongly believed that pharmacists have knowledge and skills required to deliver pharmaceutical care and providing pharmaceutical care requires special areas, 70.5% and 69.2%, respectively. The majority of study participants (80.8%) strongly agreed that providing pharmaceutical care will increase the patient's confidence in the profession. Almost three-fourths of the study participants strongly disagreed on the statement stated as PC is not the pharmacists' duty; hence, there is no need for pharmacists' involvement. The median score (with IQR) of the attitude of the participants about PC was 8.00 (7.00–9.00). In general, more than half (52.6%) of the pharmacists had favorable attitudes toward pharmaceutical care provision.

### 3.4. Factors Associated with Attitude and Knowledge of Pharmacists of Pharmaceutical Care

In the present study, we evaluated variables associated with knowledge and attitude of pharmaceutical care using Fisher's exact test. Age, sex, practice setting, years of experiences, and training curriculum were considered for possible association with pharmacists' knowledge and attitude toward pharmaceutical care. Accordingly, the training curriculum of the participants showed an association with the attitude of pharmacists (*P* value = 0.022). Similarly, pharmacists' knowledge was associated with their practice setting (*P* value = 0.008) (Table 4).

## 4. Discussion

This study is the first of its kind to assess the knowledge and attitudes of pharmacists in Ethiopia toward PC. In general, pharmacists in this study had good knowledge of PC. This finding is concordant with the studies conducted in Nigeria [23], Qatar [24], Jordan [25], and Saudi Arabia [19] but discordant with a study done in Brazil [20]. According to the latter report, community pharmacists in Brazil were more likely to provide traditional pharmacy services than clinical services such as PC, because of pressure from pharmacy owners. In addition, the inconsistency in the result might be due to the difference in the practice setting; i.e., our study involved pharmacists working in hospital and community pharmacies. Interestingly, our study showed the association between the practice setting of pharmacy professionals and knowledge of PC (*P* value = 0.008). This is consistent with a study reported by Haji et al. [24] that evaluated Qatar pharmacists' understanding and attitudes and perceived barriers related to providing PC.

Regarding attitude, the present study showed that more than half (52.6%) of the pharmacists had positive attitudes toward PC. This figure is lower than studies done in New Zeeland, Turkey, and Jordan that reported that 60% [26], 78.9% [21], and 90% [25] of the respondents had a positive attitude toward PC, respectively. Since PC practice is in its infantile stage in Ethiopia, more than half of the respondents had a fear about the feasibility of PC practice; that is why 55.2% of pharmacists thought that PC is the pharmacist's duty but it cannot be practiced feasibly. This figure was a bit higher than what was reported in a similar study done in Turkey, where 21% [25] of pharmacists agreed that PC is the pharmacist's duty but it cannot be practiced feasibly. Moreover, the majority of pharmacists (93.6%) agreed that pharmacists' opinions must be taken when establishing standards of PC in the modification of related law. This was in line with a similar study done in Saudi Arabia [19].

Meanwhile, in this study, it was noted that the training curriculum and attitude of the pharmacists showed an association (*P*=0.022). This might be due to the introduction of clinical and pharmaceutical care course as well as clerkship attachments in the revised patient-oriented Bachelor of Pharmacy (B.Pharm) curriculum. A study from the Northern part of Ethiopia also revealed the impact of clinical clerkship courses on students' attitudes toward pharmaceutical care [27]. The association between attitude and training curriculum was also supported by a study conducted outside Ethiopia [28].

The strength of this study is that, unlike other studies, it found the summary index of knowledge and attitude of both hospital and community pharmacists toward PC. The main limitation of this study is that there were small numbers of hospital and community pharmacies in Harar and Dire Dawa and its nonprobability sampling technique, and the finding could not be representative of the situation in the whole country. Therefore, larger studies to be conducted in multiple sites are needed.

## 5. Conclusion

The majority of pharmacists are knowledgeable about PC. However, nearly half of the pharmacists had an unfavorable attitude toward PC. Since unfavorable attitudes of pharmacists themselves toward PC have served as barriers for implementing PC, Harari Regional and Dire Dawa City Health Bureaus should organize and provide in-service training on PC to pharmacists working in community and hospital pharmacies. Furthermore, the bureaus should advocate PC as one area in a continuous professional development program.

## Figures and Tables

**Table 1:** Sociodemographic characteristics of pharmacists in Eastern Ethiopia, 2018 (*n* = 78).

Variables	Frequency	(%)
Age		
≥30	35	44.9
<30	43	55.1

Gender		
Male	65	88.3
Female	13	16.7

Religion		
Christian	52	66.7
Muslim	26	33.3

Current marital status		
Single	48	61.5
Married	30	38.5

Ethnicity		
Amhara	36	46.2
Oromo	33	42.3
Harari	5	6.4
Others^#^	4	5.1

Training status		
B.Pharm with old curriculum	43	55.1
B.Pharm with new clinical oriented curriculum and others^*∗*^	35	44.9

Year of experience (in years)		
<5	49	62.8
6–10	18	23.1
≥10	11	14.1

Practice setting		
Community pharmacy	34	43.6
Hospital pharmacy	44	56.4

^#^Guragae (2) and Somali (2). ^*∗*^Including B.Pharm with old curriculum plus 1 month in-service and MSc in clinical pharmacy.

**Table 2:** Knowledge of pharmacists on pharmaceutical care in Eastern Ethiopia, 2018 (*n* = 78).

No	Knowledge Assessment Question	Yes, *n* (%)	No, *n* (%)	I do not know, *n* (%)
1	PC is defined as a patient-centered way to deliver medication management services	74 (94.9)	3 (3.8)	1 (1.3)
2	PC is a philosophy of practice where pharmacists work with and for the patient to optimize the outcomes of medication therapy	74 (94.9)	3 (3.8)	1 (1.3)
3	PC stresses a pharmacist's responsibility for a patient's drug-related needs and being held accountable for the commitment	69 (88.5)	8 (10.3)	1 (1.3)
4	The purpose of PC is to achieve positive patient outcomes	76 (97.4)	2 (2.6)	0 (0)
5	Primary focus of PC in the health care system is identifying and meeting patient's drug-related needs	68 (87.2)	8 (10.3)	2 (2.6)
6	Primary responsibility of PC in the drug use process is the identification, prevention, and resolution of drug therapy problems	71 (91.0)	6 (7.7)	1 (1.3)
7	PC practitioner conducts an assessment of the patient, his/her medical problems, and drug therapies leading to drug therapy problem identification	68 (87.2)	9 (11.5)	1 (1.3)
8	PC practitioner develops a plan that establishes the desired goals of therapy for each of the patient's medical conditions	75 (96.2)	3 (3.2)	0 (0)
9	PC practitioner schedules for follow-up with the patient to evaluate the results of pharmacotherapy's, recommendations, and other interventions	64 (82.1)	11(14.1)	3 (3.8)
10	Documentation of the care provided is among the vital elements of the pharmaceutical practice process	73 (93.6)	3 (3.8)	2 (2.6)

**Table 3:** Attitude of pharmacists' toward pharmaceutical care in Eastern Ethiopia, 2018 (*n* = 78).

No	Attitude Assessment Question	*n* (%)				
		Strongly agree	Agree	Neutral	Disagree	Strongly disagree
1	All pharmacists should provide PC services	46 (59.0)	24 (30.8)	3 (3.8)	3 (3.8)	2 (2.6)
2	Pharmacists have the knowledge and skills necessary to provide PC	55 (70.5)	19 (24.4)	2 (2.6)	0 (0)	2 (2.6)
3	Providing PC requires a special area to interview patients and advise them	54 (69.2)	15 (19.2)	6 (7.7)	0 (0)	3 (3.8)
4	Providing PC will negatively affect the relationship between the pharmacist and the physician	7 (9)	15 (19.2)	8 (10.3)	15 (19.2)	33 (44.3)
5	Providing PC will increase the patients' confidence in the profession	63 (80.8)	13 (16.7)	1 (1.3)	1 (1.3)	0 (0)
6	PC is not the pharmacists' duty; hence, there is no need for pharmacists' involvement	3 (3.8)	4 (5.1)	4 (5.1)	10 (12.8)	57 (73.1)
7	PC is the pharmacists' duty, but it cannot be practiced feasibly	18 (23.1)	25 (32.1)	14 (17.9)	9 (11.5)	12 (15.4)
8	Pharmacists' opinions must be taken when establishing standards of PC in modification of related law	60 (76.9)	13 (16.7)	4 (5.1)	1 (1.3)	0 (0)
9	Providing PC is the duty of hospital pharmacists only	4 (5.1)	10 (12.8)	10 (12.8)	11 (14.1)	43 (55.1)
10	Increasing graduates of the new patient-oriented pharmacy curriculum in Ethiopia is a threat for the former pharmacy graduates	21 (26.9)	10 (12.8)	16 (20.5)	2 (2.6)	29 (37.2)

**Table 4:** Factors associated with attitude and knowledge of pharmaceutical care among pharmacists in Eastern Ethiopia, 2018 (*n* = 78).

Variables	Category	Attitudes		Knowledge			
		Favorable	Unfavorable	*P* value	Good	Poor	*P* value
Age	≥30	13 (37.1%)	22 (51.2%)	0.257	27 (77.1%)	8 (22.9%)	0.056
	<30	22 (62.9%)	21 (48.8%)		40 (93%)	3 (7%)	
Sex	Male	30 (46.2%)	35 (53.8%)	0.763	57 (87.7%)	8 (12.3%)	0.380
	Female	5 (38.5%)	8 (61.5%)		10 (76.9%)	3 (23.1%)	
Practice setting	Hospital	24 (54.5%)	20 (45.5%)		42 (95.5%)	2 (4.5%)	0.008^*∗*^
	Community	11 (32.4%)	23 (67.6%)		25 (73.5%)	9 (26.5%)	
Year of experience	<10 years	31 (46.3%)	36 (53.7%)	0.067	58 (86.6%)	9 (13.4%)	0.649
	≥10 years	4 (36.4%)	7 (63.6%)		9 (81.8%)	2 (18.2%)	
Training curriculum	B.Pharm with old curriculum	14 (32.6%)	29 (67.4%)	0.022^*∗*^	35 (81.4%)	8 (18.6%)	0.328
	B.Pharm with new clinical oriented curriculum and others	21 (60%)	14 (40%)		32 (91.4%)	3 (8.6%)	

^*∗*^Variables with the significant association.

## Data Availability

The data used to support the findings of this study are available from the corresponding author upon request.
